# Prognostic Impacts of Hypoxic Markers in Soft Tissue Sarcoma

**DOI:** 10.1155/2012/541650

**Published:** 2012-02-20

**Authors:** Eivind Smeland, Thomas K. Kilvaer, Sveinung Sorbye, Andrej Valkov, Sigve Andersen, Roy M. Bremnes, Lill-Tove Busund, Tom Donnem

**Affiliations:** ^1^Institute of Clinical Medicine, University of Tromso, 9037 Tromso, Norway; ^2^Department of Oncology, University Hospital of North Norway, 9038 Tromso, Norway; ^3^Institute of Medical Biology, University of Tromso, 9037 Tromso, Norway; ^4^Department of Pathology, University Hospital of North Norway, 9038 Tromso, Norway

## Abstract

*Background.* We aimed to explore the prognostic impact of the hypoxia-induced factors (HIF*α*s) 1 and 2, the metabolic HIF-regulated glucose transporter GLUT-1, and carbonic anhydrase IX (CAIX) in non-gastrointestinal stromal tumor soft tissue sarcomas (non-GIST STS). *Methods.* Duplicate cores with viable tumor tissue from 206 patients with non-GIST STS were obtained and tissue microarrays were constructed. Immunohistochemistry (IHC) was used to evaluate expression of hypoxic markers. *Results.* In univariate analyses, GLUT-1 (*P* < 0.001) and HIF-2*α* (*P* = 0.032) expression correlated significantly with a poor disease-specific survival (DSS). In the multivariate analysis, however, only high expression of GLUT-1 (HR 1.7, CI 95% 1.1–2.7, *P* = 0.021) was a significant independent prognostic indicator of poor DSS. *Conclusion.* GLUT-1 is a significant independent negative prognostic factor in non-GIST STS.

## 1. Background

Adaptation of tumor cells to hypoxia is critical for tumor survival and progression [1]. Tumor hypoxia leads to resistance to radiotherapy and chemotherapy and is associated with an increased metastatic potential [2, 3]. In soft tissue sarcomas (STS) hypoxia has been found to be related to reduced disease-free survival [4] and increased cell proliferation [5]. 

Principally there are three methods of measuring hypoxia, directly *in vivo* through microelectrodes (e.g., Eppendorf pO2 Histograph), visualization of nitroimidazole compounds by PET, or in tumor tissue by studying endogenous markers of hypoxia [6]. HIF-1, GLUT-1, and CAIX have been proposed as endogenous immunohistochemical (IHC) markers of hypoxia [7, 8] although this is a matter of controversy [9, 10].

Since Semenza et al. discovered HIF-1*α* in 1992, the hypoxia inducible factors (HIFs) have been identified as key regulators of genes involved in hypoxic responses [11]. Except for differences in distribution, abundance, and expression in response to prolonged hypoxia, the regulatory characteristics are quite similar among HIFs [12–15]. IHC assessments in human cancer biopsies have found elevated levels of HIF-1*α* and/or HIF-2*α* protein in the majority of primary human cancers and their metastases [16]. Furthermore, clinical data shows that increased levels of HIF-1*α* and HIF-2*α* are associated with higher patient mortality in many human cancers [17].

Cancer cells prefer glycolysis with or without hypoxia [18]. The HIF-regulated glucose transporter GLUT-1 facilitates increased influx of glucose and is upregulated in hypoxic conditions. GLUT-1 is overexpressed in several tumors [19, 20], and increased expression of GLUT-1 appears to be correlated with a poor prognosis in a variety of tumors [21–23]. CAIX mediates the extracellular trapping of acidity. Together with GLUT-1, it is among the most critical molecules, for maintaining ATP levels with stable intracellular pH, needed for cancer cell survival [24–26]. Hypoxia-related markers are inadequately explored in STS, and the data are diverging. Hence, further knowledge on the molecular mechanisms in this tumor entity is warranted.

As the prognosis of patients with STS is still unsatisfactory [27], new therapeutic targets are highly desired. The remarkable history of imatinib in CD 117 positive GIST patients is inspiring [28]. The evaluation of potential prognostic molecular markers may help to identify both promising targets and patients in need of adjuvant treatment. As a consequence, it is important to enhance our knowledge about pivotal molecular markers with inherent and diverse significant prognostic relevance for tumor progression and survival.

We have previously reported on the prognostic impact of various angiogenic factors in sarcoma [29]. Herein, using a high-throughput TMA technique, we explore the prognostic impact of markers associated with hypoxia (HIF1*α*, HIF2*α*) and related metabolic markers (GLUT-1, CAIX) in non-GIST STS.

## 2. Materials and Methods

### 2.1. Patients and Clinical Samples

 Primary tumor tissues from anonymized patients diagnosed with non-GIST STS at the University Hospital of North Norway and the Hospitals of Arkhangelsk county, Russia, from 1973 through 2006, were collected. In total 496 patients were registered from the hospital databases. Of these 290 patients were excluded from the study because of: missing clinical data (*n* = 86), inadequate paraffin-embedded fixed tissue blocks (*n* = 161), or metastasis at the time of diagnosis (*n* = 43). Thus 206 patients were included in this study.

This report includes followup data as of September 2009. The median follow-up was 37.6 (range 0.1–391.7) months. Complete demographic and clinical data were collected retrospectively. Formalin-fixed and paraffin-embedded tumor specimens were obtained from the archives of the Departments of Pathology at the University Hospital of North Norway and the Hospitals of Arkhangelsk county, Russia. The tumors were graded according to the French Fédération Nationale des centres de Lutte Contre le Cancer (FNCLCC) system and histologically subtyped according to the World Health Organization guidelines [30, 31]. Wide resection margins were defined as wide local resection with free microscopic margins or amputation of the affected limb or organ. Nonwide resection margins were defined as marginal or intralesional resection margins, or no surgery.

### 2.2. Microarray Construction

All sarcomas were histologically reviewed by two trained pathologists (S. Sorbye and A. Valkov), and the most representative areas of tumor cells (neoplastic mesenchymal cells) were carefully selected and marked on the hematoxylin and eosin (H/E) slide and sampled for the tissue microarray (TMA) blocks. The TMAs were assembled using a tissue-arraying instrument (Beecher Instruments, Silver Springs, MD). The detailed methodology has been previously reported [32]. Briefly, we used a 0.6 mm diameter stylet, and the study specimens were routinely sampled with duplicate cores from different areas of neoplastic tissue. Normal soft tissues were used as staining controls.

To include all core samples, 12 TMA blocks were constructed. Multiple 4 *μ*m sections were cut with a Micron microtome (HM355S) and stained by specific antibodies for IHC analysis.

### 2.3. Immunohistochemistry

All applied antibodies had been subjected to in-house validation by the manufacturer for IHC on paraffin-embedded material. All sections were deparaffinised with xylene and rehydrated with ethanol. The 4 *μ*m sections, containing tissue cores, were subjected to the following antibodies: HIF1*α* (mouse monoclonal, NB100-131, Novus Biologicals,1 : 3500), HIF2*α* (rabbit polyclonal, ab199, Abcam,1 : 40), GLUT-1 (mouse monoclonal, AB40084, Abcam,1 : 500), and CAIX (rabbit polyclonal, ab15086, Abcam,1 : 200).

CAIX, GLUT-1, and the HIFs were stained using the Ventana Benchmark XT (Ventana Medical Systems Inc.), procedure ultraview DAB. Antigen retrieval was done automatic by Cell Conditioning Solution (CC1) mild (30 min for CAIX and HIFs and 1 hour for GLUT-1).

The primary antibody was visualized by adding a secondary antibody conjugated with Biotin, followed by an Avidin/Biotin/Peroxydase complex (Vectastain ABC Elite kit from Vector Laboratories). Finally, all slides were counterstained with hematoxylin to visualize the nuclei.

### 2.4. Scoring of Immunohistochemistry

**Figure 1:**
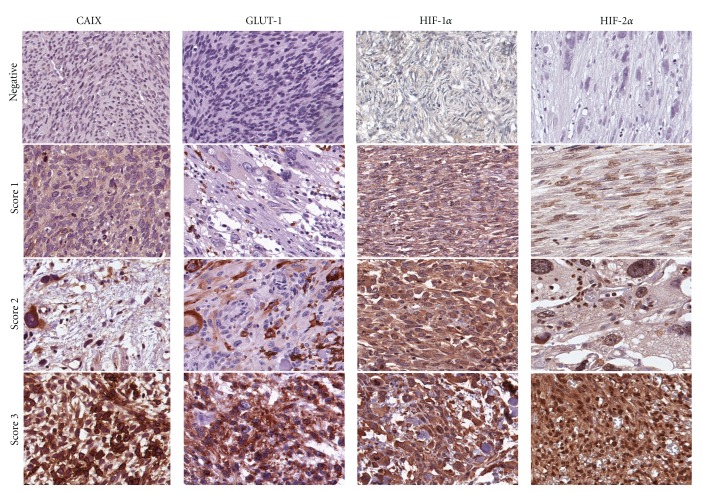
Immunohistochemical analysis in non-GIST STS representing negative, and score 1–3 of CAIX, GLUT-1, HIF-1*α*, and HIF-2*α*. non-GIST STS: non-gastrointestinal stromal tumor soft-tissue sarcomas, CAIX: carbonic anhydrase IX, GLUT-1: glucose transporter-1, and HIF-1/2*α*: hypoxia induced factor 1/2*α*.

 The ARIOL imaging system (Genetix, San Jose, CA) was used to scan the slides of antibody staining of the TMAs. The slides were loaded in the automated slide loader (Applied Imaging SL 50), and the specimens were scanned at low resolution (1.25x) and high resolution (20x) using the Olympus BX 61 microscope with an automated platform (Prior). Representative and viable tissue sections were scored manually and semiquantitatively on the computer screen. HIF-1*α* showed in most cases cytoplasmic staining or cytoplasmic and nuclear staining. For HIF-2*α* nuclear, or nuclear and weak cytoplasmic staining was recorded. Although it is suggested that nuclear HIF is the active form, it is synthesized and degraded in the cytoplasm. Hence, there may be some redistribution explaining both nuclear and cytoplasmic staining. However, the overall expression indicates upregulation of the pathway [16, 33]. GLUT-1 and CAIX antibodies usually recognize membrane-bound proteins [34–36], at least on cells of epithelial origin. But in our study, GLUT-1 showed cytoplasmic staining, and in a few cases both cytoplasmic and nuclear staining. For CAIX cytoplasmic staining was evaluated (Figure 1). Whether this is due to true differences between sarcomas and epithelial tumors, or that sarcoma cells with scanty cytoplasm render the identification of membrane staining difficult on the background of a strong cytoplasmic reactivity, remains unclear [37–39].

The dominant staining intensity was scored as: 0 = negative; 1 = weak; 2 = intermediate; 3 = strong. All samples were anonymized and independently scored by two trained pathologists (A. Valkov and S. Sorbye). When assessing a variable for a given core, the observers were blinded to the scores of the other variables and to outcome.

In case of disagreement the slides were reexamined, and consensus was reached by the observers. Mean score for duplicate cores from each individual was calculated separately. High expression was defined as: = 3 for HIF1*α*; ≥2.5 for HIF2*α*; ≥2 for CAIX; ≥1 for GLUT-1 (Figure 1).

### 2.5. Statistical Methods

All statistical analyses were done using the statistical package SPSS (Chicago, IL), version 15. The IHC scores from each observer were compared for interobserver reliability by use of a two-way random effect model with absolute agreement definition. The intraclass correlation coefficient (reliability coefficient) was obtained from these results. The Chi-square and Fishers Exact tests were used to examine the association between molecular marker expression and various clinicopathological parameters. Fisher Exact test was used when there was a 2 × 2 table, and the sample size was small (less than 5 in a given cell). Otherwise chi-square was used. We consider *r* > 0.2 as potentially relevant and due to multiple testing significant *P* value was set at <0.01 in correlation analyses. 

Univariate analyses were done using the Kaplan-Meier method, and statistical significance between survival curves was assessed by the log rank test. The significance level used for log rank test was *P* < 0.05. DSS was determined from the date of diagnosis to the time of cancer-related death. To assess the independent value of different pretreatment variables on survival, in the presence of other variables, multivariate analyses were carried out using the Cox proportional hazards model. Only variables of significant value from the univariate analyses were entered into the Cox regression analyses. Probability for stepwise entry and removal was set at .05 and .10, respectively.

## 3. Ethical Clearance

The National Data Inspection Board and The Regional (Northern Norway) Committee for Research Ethics approved the study. The committee classified the project as retrospective nontherapeutic, bio- and genetechnology science on already registered data and archived tumor material and hence specifically waived the need for consent.

## 4. Results

### 4.1. Clinicopathological Variables

The clinicopathological variables are summarized in Table 1. The median age was 60 (range 0–91) years, 57% were female, 140 patients were Norwegian, and 66 Russian. The non-GIST STSs comprised 206 tumors including angiosarcoma (*n* = 10), fibrosarcoma (*n* = 16), leiomyosarcoma (*n* = 48), liposarcoma (*n* = 32), undifferentiated pleomorphic sarcoma (*n* = 54), neurofibrosarcoma/malignant peripheral nerve sheath tumor (MPNST, *n* = 9), rhabdomyosarcoma (*n* = 12), synovial sarcoma (*n* = 13), and unspecified sarcoma (*n* = 12). The tumor origins were distributed as follows: 38% extremities, 19% trunk, 15% retroperitoneal, 8% head/neck, and 20% visceral.

### 4.2. Interobserver Variability

Interobserver scoring agreement was tested for GLUT-1. The intraclass correlation coefficient (*r*) was 0.88 (*P* < 0.001), indicating good reproducibility between the investigators.

### 4.3. Expression of Hypoxia-Related Markers and Their Correlations

None of the markers correlated significantly with age, gender, histological subgroup, tumor depth, or tumor size. High GLUT-1 expression was significantly associated (*r* = 0.35, *P* < 0.001) with a high histological grade (high expression: grade I 21.8%, grade II 39.5%, grade III 66.1%). The same significant association (*r* = 0.23, *P* = 0.001) was also found for high HIF-2*α* expression and histological grade (high expression: grade I 49.1%, grade II 71.3%, Grade III 77.3%). Futhermore, there was a significant correlation between HIF-1*α* and HIF-2*α* (*r* = 0.27, *P* < 0.001), but no other significant association was found among the markers (Table 2).

### 4.4. Univariate Analyses

Table 1 summarizes the prognostic impact of the clinicopathological variables. Age (*P* = 0.030), patient nationality (*P* = 0.014), histological entity (*P* = 0.003), tumor size (*P* = 0.019), malignancy grade (*P* < 0.001), tumor depth (*P* = 0.002), surgery (*P* < 0.001), and surgical margins (*P* < 0.001) were significant prognostic indicators for DSS.

**Figure 2:**
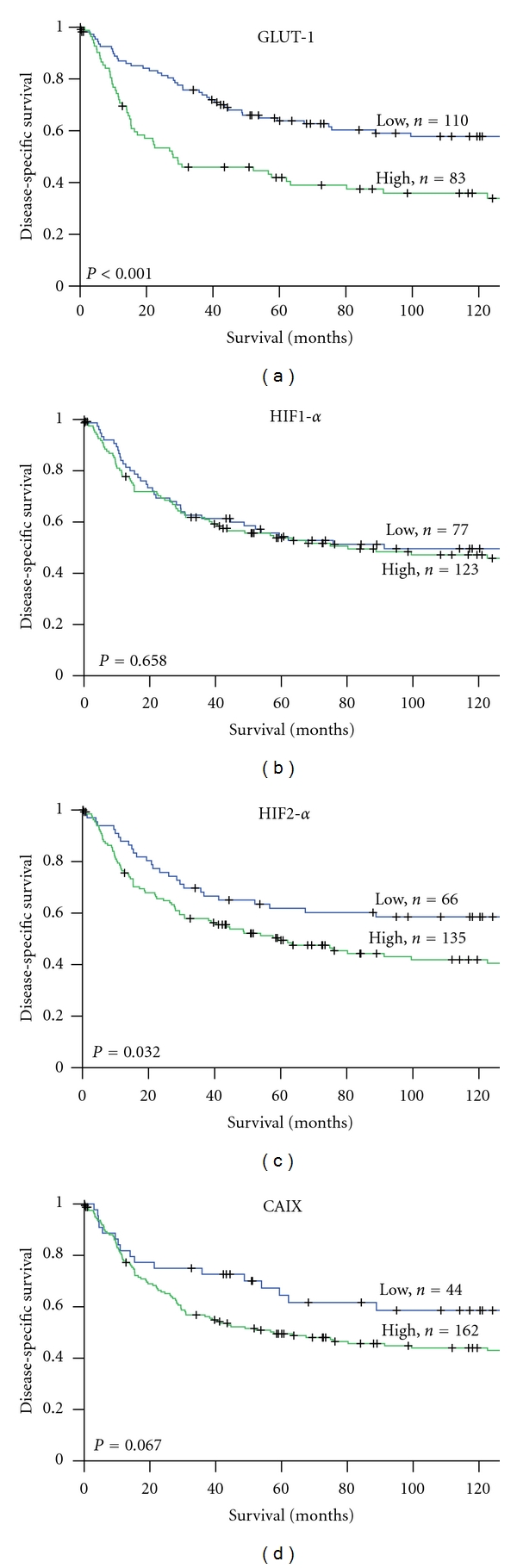
DSS curves according to GLUT-1 (a), HIF-1*α* (b), HIF-2*α* (c) and CAIX (d) expression in non-GIST STS. DSS: disease-specific survival, GLUT-1: glucose transporter-1, HIF-1/2*α*: hypoxia induced factor 1/2*α*, CAIX: carbonic anhydrase IX, and non-GIST STS: non-gastrointestinal stromal tumor soft tissue sarcomas.

Among the examined molecular markers, high tumor cell GLUT-1 expression (*P* < 0.001) and HIF-2*α* expression (*P* = 0.021) correlated significantly with a poor DSS (Table 3 and Figure 2).

Subgroup analyses with respect to histology and resection margins were done for all markers, but due to a low number of cases many results only tended to be significant. However, GLUT1 became a significant prognostic marker in pleomorphic sarcomas (*P* = 0.02). The prognostic impact of GLUT1 was not statistically significant in patients with nonwide resection margins, but significant in patients with wide resection margins (*P* = 0.001).

### 4.5. Multivariate Cox Proportional Hazards Analyses

Results from the multivariate analysis are presented in Tables 1 and 3. High malignancy grade (*P* < 0.001), deep tumor depth (*P* = 0.045), no surgery (*P* < 0.001), nonwide resection margins (*P* < 0.001), and high GLUT-1 expression were significant independent negative prognostic indicators of DSS.

## 5. Discussion

In this large-scale non-GIST STSs TMA analysis, we investigated the prognostic impact of HIF-1*α*, HIF-2*α*, and the metabolic HIF-regulated GLUT-1 and CAIX. Interestingly, high GLUT-1 expression is an independent negative prognostic factor in non-GIST STSs, while high HIF-2*α* expression is significantly associated with a poor prognosis in univariate analyses.

GLUT-1 is extensively expressed in several tumors [19, 20]. Furthermore, GLUT-1 has been correlated with a dismal prognosis in different cancer types, such as ovarial cancer [21], nonsmall cell lung cancer (NSCLC) [23], and colorectal cancer [22]. With respect to sarcoma, Ahrens et al. reported immunohistochemical GLUT-1 expression in 247 soft tissue and bone neoplasms [37]. GLUT-1 expression was seen in a wide variety of both benign and malignant mesenchymal tumors although they did not assess the prognostic impact of these markers.

Also in sarcomas has the function of GLUT-1 been related to glucose metabolism. In a prospective evaluation using a [^18^F]fluorodeoxyglucose positron emission tomography (FDG-PET), Tateishi and coworkers observed that GLUT-1 expression and enhanced glucose metabolism were associated with tumour grade in bone and soft tissue sarcomas [39]. GLUT-1-positive tumors had significantly higher mean and maximal standardized uptake values (SUVs) than the GLUT-1-negative tumors. Likewise, Nagamatsu et al. reported the use of FDG-PET for diagnosis of uterine sarcomas [40]. They detected GLUT-1 expression scores to be significantly higher in sarcomas and endometrial cancer than in leiomyomas and concluded that immunohistochemical examination of GLUT-1 confirmed the high FDG uptake in leiomyosarcoma patients.

The prognostic impact of GLUT-1 has been documented by Endo and coworkers [38]. They reported GLUT-1 expression in 22 patients with bone sarcomas and 45 with STS. They found GLUT-1 overexpression in as much as 83% of the patients. The patients with GLUT-1 overexpression showed significantly worse OS compared with those without (*P* = 0.029). Possibly due to the small number of cases, GLUT-1 did not appear as an independent prognostic factor in their study. Herein we document for the first time that GLUT-1 expression is an independent indicator of poor prognosis in non-GIST STSs.

Overexpression of HIF-2*α* was associated with reduced survival in the univariate analysis. To our knowledge, this is the first report to examine HIF-2*α* in sarcoma patients. These results are, however, comparable with findings in other tumors. Koukourakis and colleagues found that HIF-2*α* and CAIX were associated with radiotherapy failure in head and neck cancer patients [33]. Likewise, HIF-1*α* and HIF-2*α* were highly expressed in metastatic gastric cancers and correlated significantly with clinical stage [41].

In our study, HIF-1*α* overexpression was not associated with inferior survival, which is in contrast to the findings by Shintani et al. [42]. They reported IHC expression of HIF-1*α* in 49 specimens of STS and found strong and moderate HIF-1*α* expression to be independently associated with a shorter survival.

For the prognostic impact of HIF-2*α*, several reports are consistent with our results. Yoshimura et al. examined HIF-1*α* and HIF-2*α* expression in 87 resected colorectal carcinomas [43]. HIF-1*α* (45%) was more frequently expressed than HIF-2*α* (30%), but clinicopathological variables representing tumor aggressiveness correlated more often with HIF-2*α*, than HIF-1*α*. In lung cancer, Giatromanolaki and coworkers [44] found tumor HIF-2*α*-expression, not HIF-1*α*, to be independently associated with survival. Furthermore, as IHC is a “snapshot” of the tissue metabolism, involved molecules may not be expressed at high level simultaneously. HIFs are also known to be rapidly degraded [7] and differentially expressed during prolonged hypoxia [45, 46]. Other transcription factors may also be involved, hence blurring the clear-cut image we have of hypoxia pathways [47, 48].

In our study, CAIX tended towards a negative prognostic impact in the univariate analysis. In central chondrosarcoma, the only sarcoma in which CAIX has been studied, Boeuf et al. reported CAIX reactivity to be a grade-independent predictor of poor metastasis-free survival [49]. The results from other malignancies are divergent. Woelber et al. found CAIX to be upregulated in ovarian cancer [50]. Serum concentration of CAIX showed, however, no significant changes during first-line therapy, and there was no association between serum CAIX and progression-free or overall survival. In renal cell carcinoma, CAIX is strongly expressed and associated with clinical outcome, but not as an independent prognostic marker [51]. An independent prognostic impact of CAIX was, however, observed by Lie et al. in NSCLC [52]. More intriguing is the data from Eckert et al. in oral squamous cell carcinoma [53], where patients with low coexpression of HIF-1*α*/CAIX indicated a good prognosis, whereas patients with increased HIF-1*α* and low CAIX expression had around 5-fold increased risk of tumor-related death (*P* = 0.042). 

As this is a retrospective study, none of the tumors were available for *in vivo* analysis. Hence, no firm conclusions regarding these factors association to actual hypoxia itself can be drawn. *In vivo* hypoxia can only be measured directly with electrodes on tumors available for such instrumentation, indirectly by PET using nitroimidazole compounds or newer specific tracers like F-FAZA (Fluorine-fluoroazomycin arabinoside) or F-EF5 (Fluorine 2-(2-nitro-1H-imidazol-1-yl)-N-(2,2,3,3,3-pentafluoropropyl)-acetamide) [54]. In addition, it is the hypoxia *in vivo* that is clinically important since absolute hypoxia initiates when the tumor blood flow is terminated under surgery. It also has to be kept in mind that it usually takes hours until the tissues are fixed in formalin [55]. In this study we have instead explored the impact of hypoxia indirectly by evaluating proposed hypoxic markers due to their up regulation by hypoxia [8], although their role is controversial [10].

It is possible to question the heterogeneity of the patient population, regarding origin, histology, and treatment. However, this is the limitation for almost all studies on seldom malignancies such as STS. Furthermore, GLUT-1 had the same tendency in all subgroups in our material.

Another potential limitation with TMA is the eventual lack of homogeneous expression that is easily identified in larger tissue sections. Hence, to confine the impact of this issue, we used duplicate cores which were selected to be as representative as possible. Up to 95% correlation has been demonstrated when comparing tumour cell assessment in duplicate 0.6 mm cores versus the whole slide [56].

 In cancer therapy there is an increasing focus on personalized therapy. This shift is associated with the introduction of novel cytotoxic agents and molecular targeted drugs. Inhibitors of HIF-1 and CAIX have been developed and are currently under examination [57–59]. Increased knowledge about hypoxia-associated markers and metabolic markers in non-GIST-STS will be vital in identifying different tumor cell phenotypes as candidates for specific molecular targeting.

Though further studies are needed, GLUT-1 appears as a potentially relevant prognostic factor in routine examination in non-GIST-STS. The identification of independent prognostic markers in STS and other malignancies is vital for the future development of new molecular targeted drugs.

## Figures and Tables

**Table 1:** Prognostic relevance of clinicopathological variables for disease-specific survival in 206 non-gastrointestinal stromal tumor soft-tissue sarcomas (univariate analyses, log rank test, multivariate analyses, Cox proportional hazards model).

Characteristics	Univariate analyses					Multivariate analyses*		
	Patients (*n*)	Patients (%)	Median survival (months)	5-year survival (%)	*P*	HR	95% CI	*P*
*Age*					0.030			0.121^#^
≤ 20 years	17	8	41	47		1.000		
21–60 years	89	43	NR	61		0.728	0.281–1.889	0.515
>60 years	100	49	52	46		1.222	0.445–3.356	0.698
*Gender*					0.265			
Male	89	43	NR	55				
Female	117	57	75	51				
*Patient nationality*					0.014			
Norwegian	140	68	NR	58		1.000		0.142
Russian	66	32	39	42		1.453	0.882–2.393	
*Histological entity*					0.003			0.086^#^
Pleomorphic sarcoma	54	26	54	48		1.000		
Leiomyosarcoma	48	23	89	64		0.595	0.327–1.082	0.089
Liposarcoma	32	16	NR	71		0.411	0.169–0.999	0.050
Fibrosarcoma	16	8	123	56		0.773	0.330–1.808	0.552
Angiosarcoma	10	5	10	30		1.124	0.386–3.276	0.830
Rhabdomyosarcoma	12	6	41	50		0.419	0.156–1.126	0.085
MPNST	9	4	NR	56		0.544	0.162–1.824	0.324
Synovial sarcoma	13	6	31	28		1.257	0.571–2.767	0.570
Sarcoma NOS	12	6	9	25		1.640	0.707–3.805	0.249
*Tumor localization*					0.805			
Extremities	79	38	123	56				
Trunk	40	19	44	49				
Retroperitoneum	30	15	57	47				
Head/neck	16	8	27	47				
Visceral	41	20	75	57				
*Tumor size*					0.019			0.061^#^
≤5 cm	60	29	NR	66		1.000		
5–10 cm	77	37	62	51		1.478	0.825–2.646	0.189
>10 cm	67	33	37	44		2.072	1.129–3.801	0.019
Missing	2	1						
*Malignancy grade*					<0.001			<0.001^#^
1	56	27	NR	78		1.000		
2	82	40	62	51		2.761	1.352–5.639	0.005
3	68	33	22	34		4.642	2.219–9.710	<0.001
*Tumor depth*					0.002			
Superficial	16	8	NR	93		1.000		
Deep	190	92	59	50		7.658	1.043–56.215	0.045
*Surgery*					<0.001			
Yes	194	94	123	56		1.000		
No	12	6	4	0		16.689	5.776–48.218	<0.001
Resection margins					<0.001			
Wide	97	47	NR	66		1.000		
Nonwide/no surgery	109	53	36	41		1.894	1.214–2.955	0.005
*Chemotherapy *					0.641			
No	166	81	91	54				
Yes	40	19	41	47				
*Radiotherapy*					0.224			
No	141	69	127	55				
Yes	65	32	52	49				

NR: not reached; MPNST: malignant peripheral nerve sheath tumor; NOS: not otherwise specified

*Only significant variables from the univariate analyses were entered into the multivariate analyses

^#^Overall significance as prognostic factor.

**Table 2:** Correlation between expression of hypoxic markers and clinicopathological variables.

	GLUT-1	HIF-1*α*	HIF-2*α*	CAIX
GLUT-1		*r* = 0.08	*r* = 0.11	*r* = 0.18
		*P* = 0.29	*P* = 0.12	*P* = 0.013
HIF-1*α*	*r* = 0.08		**r** = 0.27	*R* = 0.18
	*P* = 0.29		**P** < 0.001	*P* = 0.013
HIF-2*α*	*r* = 0.11	**r** = 0.27		*r* = 0.17
	*P* = 0.12	**P** < 0.001		*P* = 0.017
CAIX	*r* = 0.18	*r* = 0.18	*r* = 0.17	
	*P* = 0.013	*P* = 0.013	*P* = 0.017	
Age	*r* = 0.12	*r* = 0.15	*r* = −0.04	*r* = −0.02
	*P* = 0.11	*P* = 0.93	*P* = 0.58	*P* = 0.74
Gender	*r* = 0.13	*r* = −0.001	*r* = 0.07	*r* = 0.10
	*P* = 0.07	*P* = 0.97	*P* = 0.30	*P* = 0.17
Patient nationality	*r* = −0.03	*r* = −0.15	*r* = 0.17	*r* = 0.001
	*P* = 0.67	*P* = 0.03	*P* = 0.02	*P* = 0.97
Histological entity	*r* = 0.08	*r* = −0.05	*r* = −0.06	*r* = −0.03
	*P* = 0.30	*P* = 0.49	*P* = 0.43	*P* = 0.66
Tumor localization	*r* = 0.09	*r* = −0.02	*r* = 0.03	*r* = 0.19
	*P* = 0.23	*P* = 0.74	*P* = 0.69	*P* = 0.006
Tumor size	*r* = 0.04	*r* = −0.004	*r* = 0.01	*r* = −0.01
	*P* = 0.59	*P* = 0.95	*P* = 0.85	*P* = 0.92
Malignancy grade	**r** = 0.35	*r* = −0.01	**r** = 0.23	*r* = 0.13
	**P** < 0.001	*P* = 0.85	**P** = 0.001	*P* = 0.06
Tumor depth	*r* = 0.10	*r* = 0.07	*r* = −0.05	*r* = 0.03
	*P* = 0.19	*P* = 0.33	*P* = 0.49	*P* = 0.71
Resection margins	*r* = −0.10	*r* = −0.13	*r* = −0.13	*r* = −0.05
	*P* = 0.18	*P* = 0.06	*P* = 0.07	*P* = 0.49

*r* = correlation coefficient.

**Table 3:** Tumor expression GLUT-1, HIF-1*α*, HIF-2*α*, and CAIX and their prognostic relevance for disease-specific survival in 206 patients with non-gastrointestinal soft tissue (univariate analyses; log-rank test, multivariate analyses; Cox proportional hazards model).

Characteristics	Univariate analyses					Multivariate analyses*		
	Patients (*n*)	Patients (%)	Median survival (months)	5-year survival (%)	*P*	HR	95% CI	*P*
*GLUT-1*					<0.001			
Low expression	110	53	NR	64		1.000		
High expression	83	40	28	42		1.697	1.083–2.659	0.021
Missing	13	6						
*HIF-1*α**					0.658			
Low expression	77	37	91	54				
High expression	123	60	80	54				
Missing	6	3						
*HIF-2*α**					0.032			
Low expression	66	32	NR	62		1.000		
High expression	135	66	59	49		0.965	0.581–1.603	0.892
Missing	5	2						
*CAIX*					0.067			
Low expression	44	21	NR	65				
High expression	162	79	58	50				

NR: not reached.

*Only significant variables from the univariate analyses were entered into the multivariate analyses.

## References

[1] Pouysségur J, Dayan F, Mazure NM (2006). Hypoxia signalling in cancer and approaches to enforce tumour regression. *Nature*.

[2] Airley RE, Phillips RM, Evans AE (2005). Hypoxia-regulated glucose transporter Glut-1 may influence chemosensitivity to some alkylating agents: results of EORTC (First Translational Award) study of the relevance of tumour hypoxia to the outcome of chemotherapy in human tumour-derived xenografts. *International Journal of Oncology*.

[3] Hockel M, Schlenger K, Aral B, Mitze M, Schaffer U, Vaupel P (1996). Association between tumor hypoxia and malignant progression in advanced cancer of the uterine cervix. *Cancer Research*.

[4] Brizel DM, Scully SP, Harrelson JM (1996). Tumor oxygenation predicts for the likelihood of distant metastases in human soft tissue sarcoma. *Cancer Research*.

[5] Nordsmark M, Hoyer M, Keller J, Nielsen OS, Jensen OM, Overgaard J (1996). The relationship between tumor oxygenation and cell proliferation in human soft tissue sarcomas. *International Journal of Radiation Oncology Biology Physics*.

[6] Mortensen LS, Buus S, Nordsmark M (2010). Identifying hypoxia in human tumors: a correlation study between 18F-FMISO PET and the Eppendorf oxygen-sensitive electrode. *Acta Oncologica*.

[7] Kaluz S, Kaluzová M, Liao SY, Lerman M, Stanbridge EJ (2009). Transcriptional control of the tumor- and hypoxia-marker carbonic anhydrase 9: a one transcription factor (HIF-1) show?. *Biochimica et Biophysica Acta, Reviews on Cancer*.

[8] Vordermark D, Brown JM (2003). Endogenous markers of tumor hypoxia: predictors of clinical radiation resistance?. *Strahlentherapie und Onkologie*.

[9] Jankovic B, Aquino-Parsons C, Raleigh JA (2006). Comparison between pimonidazole binding, oxygen electrode measurements, and expression of endogenous hypoxia markers in cancer of the uterine cervix. *Cytometry Part B, Clinical Cytometry*.

[10] Mayer A, Hockel M, Vaupel P (2008). Endogenous hypoxia markers: case not proven!. *Advances in Experimental Medicine and Biology*.

[11] Semenza GL, Wang GL (1992). A nuclear factor induced by hypoxia via de novo protein synthesis binds to the human erythropoietin gene enhancer at a site required for transcriptional activation. *Molecular and Cellular Biology*.

[12] Tian H, McKnight SL, Russell DW (1997). Endothelial PAS domain protein 1 (EPAS1), a transcription factor selectively expressed in endothelial cells. *Genes and Development*.

[13] Heidbreder M, Frohlich F, Johren O, Dendorfer A, Qadri F, Dominiak P (2003). Hypoxia rapidly activates HIF-3alpha mRNA expression. *The FASEB Journal*.

[14] Gu YZ, Moran SM, Hogenesch JB, Wartman L, Bradfield CA (1998). Molecular characterization and chromosomal localization of a third *α*- class hypoxia inducible factor subunit, HIF3*α*. *Gene Expression*.

[15] Ema M, Taya S, Yokotani N, Sogawa K, Matsuda Y, Fujii-Kuriyama Y (1997). A novel bHLH-PAS factor with close sequence similarity to hypoxia-inducible factor 1*α* regulates the VEGF expression and is potentially involved in lung and vascular development. *Proceedings of the National Academy of Sciences of the United States of America*.

[16] Zhong H, de Marzo AM, Laughner E (1999). Overexpression of hypoxia-inducible factor 1{{*α*}} in common human cancers and their metastases. *Cancer Research*.

[17] Semenza GL (2010). Defining the role of hypoxia-inducible factor 1 in cancer biology and therapeutics. *Oncogene*.

[18] Warburg O (1956). On the origin of cancer cells. *Science*.

[19] Kunkel M, Reichert TE, Benz P (2003). Overexpression of Glut-1 and increased glucose metabolism in tumors are associated with a poor prognosis in patients with oral squamous cell carcinoma. *Cancer*.

[20] Younes M, Lechago LV, Somoano JR, Mosharaf M, Lechago J (1996). Wide expression of the human erythrocyte glucose transporter Glut1 in human cancers. *Cancer Research*.

[21] Cantuaria G, Fagotti A, Ferrandina G (2001). GLUT-1 expression in ovarian carcinoma: association with survival and response to chemotherapy. *Cancer*.

[22] Haber RS, Rathan A, Weiser KR (1998). GLUT1 glucose transporter expression in colorectal carcinoma: a marker for poor prognosis. *Cancer*.

[23] Younes M, Brown RW, Stephenson M, Gondo M, Cagle PT (1997). Overexpression of Glut1 and Glut3 in stage I nonsmall cell lung carcinoma is associated with poor survival. *Cancer*.

[24] Brahimi-Horn MC, Chiche J, Pouysségur J (2007). Hypoxia and cancer. *Journal of Molecular Medicine*.

[25] Chiche J, Ilc K, Laferrière J (2009). Hypoxia-inducible carbonic anhydrase IX and XII promote tumor cell growth by counteracting acidosis through the regulation of the intracellular pH. *Cancer Research*.

[26] Semenza GL (2009). Regulation of cancer cell metabolism by hypoxia-inducible factor 1. *Seminars in Cancer Biology*.

[27] Jemal A, Siegel R, Ward E, Hao Y, Xu J, Thun MJ (2009). Cancer statistics, 2009. *CA Cancer Journal for Clinicians*.

[28] Blay JY, von Mehren M, Blackstein ME (2010). Perspective on updated treatment guidelines for patients with gastrointestinal stromal tumors. *Cancer*.

[29] Kilvaer TK, Valkov A, Sorbye S (2010). Profiling of VEGFs and VEGFRs as prognostic factors in soft tissue sarcoma: VEGFR-3 is an independent predictor of poor prognosis. *PLoS One*.

[30] Fletcher CDM (2006). The evolving classification of soft tissue tumours: an update based on the new WHO classification. *Histopathology*.

[31] Guillou L, Coindre JM, Bonichon F (1997). Comparative study of the National Cancer Institute and French Federation of Cancer Centers Sarcoma Group grading systems in a population of 410 adult patients with soft tissue sarcoma. *Journal of Clinical Oncology*.

[32] Donnem T, Al-Saad S, Al-Shibli K (2007). Inverse prognostic impact of angiogenic marker expression in tumor cells versus stromal cells in non-small cell lung cancer. *Clinical Cancer Research*.

[33] Koukourakis MI, Bentzen SM, Giatromanolaki A (2006). Endogenous markers of two separate hypoxia response pathways (hypoxia inducible factor 2 alpha and carbonic anhydrase 9) are associated with radiotherapy failure in head and neck cancer patients recruited in the CHART randomized trial. *Journal of Clinical Oncology*.

[34] Choi YS, Kim SJ, Kim DS (2007). Glucose transporter-1 expression in squamous cell carcinoma of the tongue. *Cancer Research and Treatment*.

[35] Ozbudak IH, Shilo K, Tavora F (2009). Glucose transporter-1 in pulmonary neuroendocrine carcinomas: expression and survival analysis. *Modern Pathology*.

[36] Woelber L, Kress K, Kersten JF (2011). Carbonic anhydrase IX in tumor tissue and sera of patients with primary cervical cancer. *BMC Cancer*.

[37] Ahrens WA, Ridenour RV, Caron BL, Miller DV, Folpe AL (2008). GLUT-1 expression in mesenchymal tumors: an immunohistochemical study of 247 soft tissue and bone neoplasms. *Human Pathology*.

[38] Endo M, Tateishi U, Seki K (2007). Prognostic implications of glucose transporter protein-1 (Glut-1) overexpression in bone and soft-tissue sarcomas. *Japanese Journal of Clinical Oncology*.

[39] Tateishi U, Yamaguchi U, Seki K, Terauchi T, Arai Y, Hasegawa T (2006). Glut-1 expression and enhanced glucose metabolism are associated with tumour grade in bone and soft tissue sarcomas: a prospective evaluation by [18F]fluorodeoxyglucose positron emission tomography. *European Journal of Nuclear Medicine and Molecular Imaging*.

[40] Nagamatsu A, Umesaki N, Li L, Tanaka T (2010). Use of 18F-fluorodeoxyglucose positron emission tomography for diagnosis of uterine sarcomas. *Oncology Reports*.

[41] Wang Y, Li Z, Zhang H (2010). HIF-1*α* and HIF-2*α* correlate with migration and invasion in gastric cancer. *Cancer Biology and Therapy*.

[42] Shintani K, Matsumine A, Kusuzaki K (2006). Expression of hypoxia-inducible factor (HIF)-1*α* as a biomarker of outcome in soft-tissue sarcomas. *Virchows Archiv*.

[43] Yoshimura H, Dhar DK, Kohno H (2004). Prognostic impact of hypoxia-inducible factors 1*α* and 2*α* in colorectal cancer patients: correlation with tumor angiogenesis and cyclooxygenase-2 expression. *Clinical Cancer Research*.

[44] Giatromanolaki A, Koukourakis MI, Sivridis E (2001). Relation of hypoxia inducible factor 1*α* and 2*α* in operable non-small cell lung cancer to angiogenic/molecular profile of tumours and survival. *British Journal of Cancer*.

[45] Li QF, Wang XR, Yang YW, Lin H (2006). Hypoxia upregulates hypoxia inducible factor (HIF)-3*α* expression in lung epithelial cells: characterization and comparison with HIF-1*α*. *Cell Research*.

[46] Uchida T, Rossignol F, Matthay MA (2004). Prolonged hypoxia differentially regulates hypoxia-inducible factor (HIF)-1*α* and HIF-2*α* expression in lung epithelial cells: implication of natural antisense HIF-1*α*. *Journal of Biological Chemistry*.

[47] Ishibashi H, Nakagawa K, Onimaru M (2000). Sp1 decoy transfected to carcinoma cells suppresses the expression of vascular endothelial growth factor, transforming growth factor/*β*1, and tissue factor and also cell growth and invasion activities. *Cancer Research*.

[48] Pore N, Gupta AK, Cerniglia GJ (2006). Nelfinavir down-regulates hypoxia-inducible factor 1*α* and VEGF expression and increases tumor oxygenation: implications for radiotherapy. *Cancer Research*.

[49] Boeuf S, Bovée JVMG, Lehner B, Hogendoorn PCW, Richter W (2010). Correlation of hypoxic signalling to histological grade and outcome in cartilage tumours. *Histopathology*.

[50] Woelber L, Mueller V, Eulenburg C (2010). Serum carbonic anhydrase IX during first-line therapy of ovarian cancer. *Gynecologic Oncology*.

[51] Leibovich BC, Sheinin Y, Lohse CM (2007). Carbonic anhydrase IX is not an independent predictor of outcome for patients with clear cell renal cell carcinoma. *Journal of Clinical Oncology*.

[52] Lie M, Mazure NM, Hofman V (2010). High levels of carbonic anhydrase IX in tumour tissue and plasma are biomarkers of poor prognostic in patients with non-small cell lung cancer. *British Journal of Cancer*.

[53] Eckert AW, Lautner MHW, Schütze A (2010). Co-expression of Hif1*α* and CAIX is associated with poor prognosis in oral squamous cell carcinoma patients. *Journal of Oral Pathology and Medicine*.

[54] Lapi SE, Voller TF, Welch MJ (2009). Positron emission tomography imaging of hypoxia. *PET Clinics*.

[55] Sauter (2010). *Tissue Microarrays*.

[56] Kallioniemi OP, Wagner U, Kononen J, Sauter G (2001). Tissue microarray technology for high-throughput molecular profiling of cancer. *Human Molecular Genetics*.

[57] Dikmen ZG, Gellert GC, Dogan P (2008). In vivo and in vitro effects of a HIF-1*α* inhibitor, RX-0047. *Journal of Cellular Biochemistry*.

[58] Semenza GL (2007). Evaluation of HIF-1 inhibitors as anticancer agents. *Drug Discovery Today*.

[59] Thiry A, Dogne JM, Masereel B, Supuran CT (2006). Targeting tumor-associated carbonic anhydrase IX in cancer therapy. *Trends in Pharmacological Sciences*.

